# Obstacles and Enablers Related to Gestational Diabetes Self-Management: Systematic Review Using the Socio-Eecological Model

**DOI:** 10.2196/86767

**Published:** 2026-06-01

**Authors:** Bronya H K Luk, Polly H X Ma, Rosenna Wai Ming Chung, Wendy W Zhang, William Wing-kuen Lam

**Affiliations:** 1 School of Nursing and Health Sciences, Hong Kong Metropolitan University, Homantin, Hong Kong, 00852, China (Hong Kong), 852 3970 2975, 852 24062375

**Keywords:** obstacles, enablers, self-management, socioecological model, gestational diabetes mellitus, GDM, digital health

## Abstract

**Background:**

Gestational diabetes mellitus (GDM) requires effective self-management to mitigate associated health risks. A comprehensive understanding of the multilevel factors influencing adherence is essential to designing effective, holistic support strategies.

**Objective:**

This systematic review aimed to synthesize the obstacles and enablers related to GDM self-management across all 5 levels of the socioecological model (SEM).

**Methods:**

A systematic search was conducted across 6 databases (MEDLINE, PsycINFO, CINAHL, PubMed, Cochrane Library, and Web of Science) for literature published from January 2010 to October 2025. Thirty studies (24 qualitative, 4 quantitative, and 2 mixed methods) were included. Data on obstacles and enablers were extracted and synthesized using the SEM as an analytical framework.

**Results:**

The analysis identified 11 key factors across the intrapersonal (eg, knowledge and emotional response), interpersonal (eg, functional support network), organizational (eg, health care access and workplace demands), community (eg, food environment and digital information landscape), and policy levels (eg, funding and economic support). The synthesis reveals how these factors interact across levels, creating systemic challenges such as fragmentation of support and information inequity.

**Conclusions:**

Successful GDM self-management support requires integrated, multilevel strategies that address factors ranging from the individual level to the policy level. This review provides an evidence-based SEM framework to inform the development of comprehensive support interventions, including those leveraging digital health platforms, to improve maternal and neonatal outcomes.

## Introduction

### Background

Gestational diabetes mellitus (GDM) refers to any degree of glucose intolerance with onset or first recognition during pregnancy [1,2]. GDM is a common and consequential health condition during pregnancy, associated with short- and long-term risks for both mother and child. Women diagnosed with GDM face a significant risk of severe health complications such as hyperglycemia, respiratory issues, macrosomia, premature birth, birth injuries, labor dystocia, and even pregnancy-induced hypertension and heart disease [3-6]. Furthermore, their children, in later development, are more likely to experience childhood obesity, early onset of type II diabetes, and an increased risk of cardiovascular disease later in life [7,8]. A cohort study conducted in a diabetes center in Hong Kong revealed that out of 238 women with GDM and postpartum impaired glucose tolerance detected during the postdelivery period, one-fifth (20%) of them developed type II diabetes after an average follow-up period of 4.3 years [9].

GDM currently affects 2% to 10% of pregnant women in the United States, 2% to 6% in Europe, 5% to 8% in Australia, and 10% to 15% in China [10,11]. Asian women, compared to women of many other ethnic backgrounds, are at a higher risk of developing GDM [11,12]. As one of the most common health problems in pregnancy, GDM requires proactive self-management, particularly through lifestyle modifications in diet and exercise, which can reduce complications for both the mother and infant [1,2,7,13].

Self-management refers to an individual’s ability to manage the symptoms, treatments, physical and psychosocial consequences, and lifestyle changes inherent to chronic disease [14,15]. GDM self-management includes self-monitoring of blood glucose, adjusting diet, and increasing physical exercise [7,12].

Self-management skills are also necessary for pregnant women to manage their perception of health conditions that may induce stress, from pregnancy to the postnatal period. Reviews have revealed that self-management resulted in statistically and clinically significant improvements in anthropometric outcomes among women with gestational diabetes [16] and provided them with greater control over their diet and body weight [13]. An interventional study on self-management in chronic disease management demonstrated improved health status, health behavior, and self-efficacy, as well as fewer emergency room visits for participants [17]. Studies report that women diagnosed with GDM face challenges in adapting to their new diagnosis [18-21]. Women often feel frustrated by being diagnosed with the condition and experience a lack of control [21-23].

A study found that categories of factors affecting self-management include personal lifestyle characteristics, health status, resources, environmental characteristics, and the health care system. Factors may interact to affect self-management and may exist on a continuum from facilitator to barrier [24]. Understanding the factors that affect self-management could enhance the evaluation of self-management among pregnant women with gestational diabetes. This understanding could also guide the development of interventions designed to address individual needs and improve health outcomes. These findings suggest that focusing solely on individual responsibility is insufficient; a broader perspective is needed to understand the multilevel system of factors influencing self-management.

### Guiding Framework: The Socioecological Model

This review emphasizes the use of the socioecological model (SEM) as a guiding framework for understanding the factors that influence self-management behaviors in women with gestational diabetes. The SEM, widely accepted and used for understanding the health behaviors of individuals, takes into account the dynamic interplay between individuals and their environments as determinants of health-related behavior [25]. This model recognizes that an individual’s behavior is influenced by multilevel factors [25,26]. Applied to this review, the SEM provides a framework to systematically identify and analyze the obstacles and enablers of GDM self-management across these levels, from personal experience to policy context.

### Aims of This Review

The aim of this systematic review is to comprehensively explore and identify the obstacles and enablers to the self-management of gestational diabetes in pregnant women. It seeks to understand the specific obstacles and enablers at various levels—intrapersonal, interpersonal, organizational, community, and policy—that influence the self-management of this condition. The review will use the SEM to provide a holistic understanding of the factors affecting the self-management of gestational diabetes among pregnant women.

## Methods

### Data Search Strategy

A comprehensive and reproducible search strategy was developed to systematically identify all relevant literature. The search terms were developed through an iterative process involving a review of key papers on GDM self-management to identify relevant terminology, consultation with database thesauri (eg, MeSH in MEDLINE) to incorporate controlled vocabulary, and the combination of 3 core conceptual categories: GDM (eg, “gestational diabetes mellitus”), self-management (eg, “self-care” and “self-monitoring”), and determinants (eg, “barriers,” “facilitators,” and “enablers”). Boolean operators (AND, OR) were used to link these concepts. The final search strategy was peer-reviewed by a university research librarian to enhance its sensitivity and specificity. Electronic databases to be systematically searched included MEDLINE, PsycINFO, CINAHL, PubMed, Cochrane Library, and Web of Science. The keywords that were searched included the following: (“gestational diabetes” OR “gestational diabetes mellitus” OR “diabetes in pregnancy” OR “GDM”) AND (“self-management” OR “self-care” OR “self-regulation” OR “self-monitoring”) AND (“barriers” OR “obstacles” OR “challenges” OR “difficulties” OR “issues” OR “problems” OR “facilitators” OR “motivators” OR “enablers”).

The initial search was conducted in February 2024 and was limited to papers published in English between 2010 and the end of 2023. To ensure the review included the most current evidence, the search was updated in October 2025 to cover publications from January 2024 to October 2025. A citation search of the included studies was also performed to identify additional relevant studies.

This study has been registered with PROSPERO (CRD42023491588) and was conducted in adherence to the PRISMA (Preferred Reporting Items for Systematic Reviews and Meta-Analyses) guidelines (Checklist 1).

### Eligibility Criteria

Studies were included if they met the following criteria: (1) the population comprised pregnant women diagnosed with GDM, excluding studies focused solely on the postpartum period; (2) the study investigated barriers, facilitators, obstacles, enablers, challenges, or motivators related to GDM self-management, which included behaviors such as dietary modification, physical activity, self-monitoring of blood glucose, and medication adherence; (3) the study was conducted in any health care or community setting; and (4) the study design was qualitative, quantitative descriptive, or mixed methods. Review papers, commentaries, conference abstracts, and protocol papers were excluded. Only studies published in English from January 2010 onward were considered, incorporating an initial search up to the end of 2023 and an updated search to October 2025.

### Study Selection

A total of 5843 records were identified through the initial database search. After removing 853 duplicates using Covidence (Veritas Health Innovation Ltd), 4990 unique studies underwent title and abstract screening, resulting in 126 reports sought for retrieval. Of these, 119 were successfully retrieved for full-text eligibility assessment. Additionally, 32 records from an updated search (2024‐2025) and 8 records from citation searching were screened, with 11 and 8 proceeding to full-text assessment, respectively. Following full-text review, 25 studies from the database search, 3 from the updated search, and 2 from the citation search met the inclusion criteria, yielding a total of 30 studies in the final synthesis. The study selection process is detailed in the PRISMA flow diagram in *Results* section. Screening and eligibility assessments were performed independently by 2 reviewers, with discrepancies resolved through discussion and, where necessary, arbitration by a third reviewer.

The initial screening of titles and abstracts was conducted independently by 2 reviewers. The full texts of potentially eligible studies were subsequently assessed for eligibility against the inclusion and exclusion criteria, also independently by both reviewers. Any discrepancies regarding the inclusion or exclusion of studies were resolved through discussion with a third reviewer until a consensus was reached. This same collaborative process was applied during the quality assessment stage.

### Quality Assessment

The methodological quality of each included study was appraised independently by 2 reviewers using the Mixed Methods Appraisal Tool (MMAT; version 2018; McGill University) [27]. Based on the study designs identified in our review, only the MMAT checklists for qualitative, quantitative, descriptive, and mixed methods studies were applied; no randomized controlled trials or nonrandomized quantitative studies were included. The complete quality assessment results are provided in Multimedia Appendix 1.

### Data Analysis and Synthesis

In this review, the terms “barriers” and “obstacles” are used interchangeably, as are the terms “facilitators” and “enablers,” to reflect the language used across the included literature. Data extraction and synthesis followed a convergent qualitative synthesis design, explicitly guided by the SEM [25] as an analytical framework. The process consisted of 3 stages: extraction, thematic synthesis, and framework analysis.

#### Stage 1: Data Extraction and Initial Coding

Two reviewers independently extracted all relevant text segments (eg, sentences and paragraphs) pertaining to barriers, facilitators, obstacles, or enablers to GDM self-management from the “Results” or “Findings” sections of each included study. These raw data segments were imported into a standardized spreadsheet alongside key study characteristics (author, year, country, and design). Through an iterative process of discussion and consensus, the reviewers inductively coded these segments, generating a preliminary list of 32 distinct factors (eg, “language barrier,” “physical discomfort,” and “emotional support”).

#### Stage 2: Inductive Thematic Synthesis

Using a constant comparative method, 2 reviewers iteratively analyzed, compared, and grouped the 32 initial factors based on conceptual similarity and shared meaning. This inductive process collapsed the factors into 11 broader, overarching themes that captured the core phenomena influencing self-management (eg, “knowledge barriers” and “language barriers” were synthesized under the theme “Disease and Self-Management Literacy”). Discrepancies in thematic grouping were resolved through consensus discussion, with a third reviewer consulted for arbitration when needed.

#### Stage 3: Framework Analysis (Mapping to the SEM)

Each of the 11 synthesized themes was mapped to 1 primary level of the SEM (intrapersonal, interpersonal, organizational, community, and policy). This mapping was performed independently by 2 reviewers using standard definitions of the SEM levels [25]. The guiding principle was to assign a theme to the level at which the factor was most directly experienced or enacted by the woman, or where the most relevant intervention point would logically exist. For instance, “lack of shared family responsibility” was classified at the interpersonal level as a dynamic within the woman’s support network, while “personal time constraints” (a related but distinct factor) was classified at the intrapersonal level as an individual logistical challenge. All mapping decisions were documented, and disagreements were resolved through discussion to ensure consistency.

#### Integration of Quantitative Findings

Quantitative data from the included descriptive studies (eg, percentages of women reporting a specific barrier) were extracted and synthesized narratively. These findings were not analyzed separately for metasynthesis but were used to triangulate, corroborate, and add magnitude to the qualitative themes during the interpretation phase, providing an indication of the prevalence of certain experiences.

### Ethical Considerations

This study does not involve human participants.

## Results

### Characteristics of the Selected Studies

**Figure 1.**
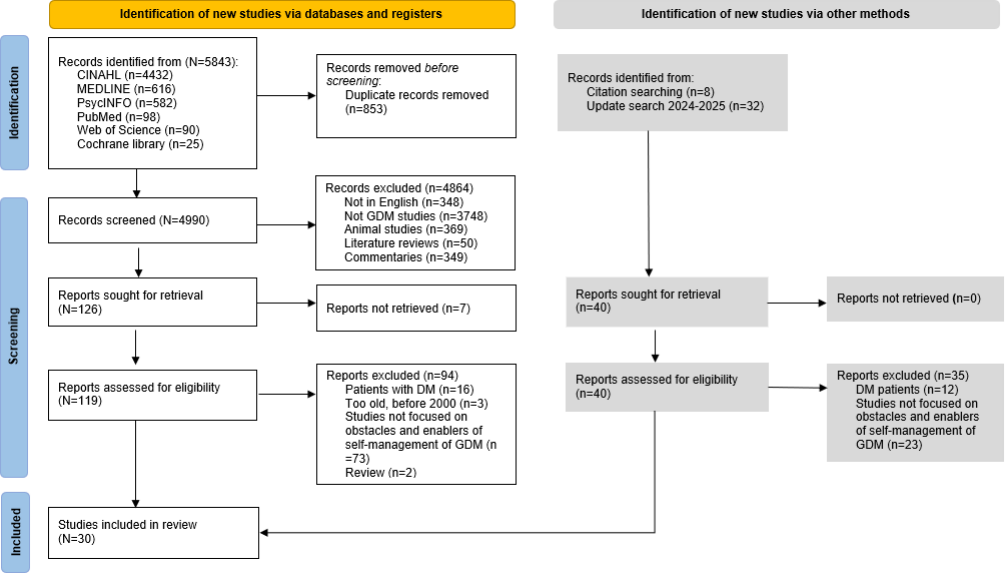
The flow diagram depicts different phases of the systematic review. DM: diabetes mellitus; GDM: gestational diabetes mellitus.

This systematic eligibility review process resulted in a final dataset of 30 studies on self-management of GDM, with 24 being qualitative studies, 4 quantitative studies, and 2 mixed methods studies (Figure 1). The identified studies represent a diverse population from 13 countries. Most were conducted in Oceania (n=13), followed by Asia (n=7), North America (n=5), Europe (n=4), and Africa (n=1). The total number of participants in these studies was 2070, with sample sizes ranging from 9 to 564.

The majority of the studies recruited participants through the traditional method of direct contact with pregnant women during clinic visits (n=20). However, newer recruitment methods were also introduced, such as email (n=4), phone (n=3), mobile applications (n=1), and social media (n=2).

In the self-management of GDM, diet modification was the most commonly adopted approach according to 27 studies, followed by exercise (n=23), blood glucose monitoring (n=20), and insulin self-administration (n=3). The characteristics of the selected studies are summarized and listed in Table 1.

**Table 1.** Characteristics of the selected studies.

Author, year, and country	Aims	Study design	Participants	Method of recruitment	Type of self-management
Jirojwong et al, 2017, Australia [28]	To explore how Southeast Asian migrant women in Australia experience and manage GDM^a^.	Qualitative	19 Southeast Asian migrant women	Clinic patients	Diet, exercise, and blood glucose monitoring
Harrison et al, 2019, Australia [29]	To determine the attitudes of women diagnosed with GDM toward physical activity during pregnancy and to identify the perceived barriers and enablers of physical activity during pregnancy in women with GDM.	Qualitative	27 individuals with diverse ethnicities	Clinic patients	Exercise
de Sequeira et al, 2019, Canada [30]	To explore, among South Asian immigrant women with GDM: (1) their awareness of diabetes education resources, (2) their attitudes toward information from different resources and varying health care providers, and (3) their barriers and facilitators for GDM management recommendations.	Qualitative	13 South Asian immigrant women	Clinic patients	Diet, exercise, and blood glucose monitoring
Skar et al, 2018, Norway [31]	To explore the experiences of women with GDM by controlling their blood glucose values and receiving health and nutrition information using a smartphone app (Pregnant+ app; Philips Digital UK Ltd).	Qualitative	17 individuals with diverse ethnicities	Phone	Diet, exercise, and blood glucose monitoring
Kaptein et al, 2015, Canada [32]	To gain insight into the reactions and experiences of multiethnic women diagnosed with GDM.	Qualitative	19 individuals with diverse ethnicities	Clinic patients	Diet and exercise
Siad et al, 2018, Canada [33]	To document the impact of a diagnosis of GDM and the perceptions of diabetes care among East African immigrant women.	Qualitative	10 East African immigrant women	Clinic patients	Diet, exercise, and blood glucose monitoring
Al Nadhiri et al, 2023, Oman [34]	To examine the predictors, the barriers, and the motivating factors of adherence to the GDM management plan among Arab pregnant women with GDM.	Cross-sectional	164 Arabian	Clinic patients	Diet, exercise, and blood glucose monitoring
Khooshehchin et al, 2016, Iran [35]	Aimed at a deeper understanding of women’s experiences of gestational diabetes and their perceived needs to inform future lifestyle interventions.	Qualitative	12 Iranian	Clinic patients	Diet and insulin self-administration
Guo et al, 2020, China [36]	To describe the current status of self-monitoring of blood glucose (SMBG) engagement among women with GDM in China and identify its barriers and facilitators.	Mixed methods	323 Chinese women completed the survey and 11 participated in the interview	Clinic patients	Diet, exercise, and blood glucose monitoring
Wah et al, 2019, Australia [37]	To explore the understanding and self-management experiences of gestational diabetes among Chinese migrants.	Qualitative	18 Chinese immigrant women	Clinic patients	Diet, exercise, and blood glucose monitoring
Smyth et al, 2023, Ireland [38]	To explore the attitudes of women with GDM toward these lifestyle changes, specifically diet and exercise. Women were also asked to identify advice that would be useful for other women newly diagnosed with GDM.	Qualitative	32 (28 Irish and European, 4 non-European)	Clinic patients	Diet and exercise
Yee et al, 2015, United States [39]	To evaluate barriers to diabetes self-care during pregnancy in an underserved population.	Qualitative	10 individuals with diverse ethnicities	Clinic patients	Diet, exercise, and blood glucose monitoring
Martis et al, 2017, New Zealand [40]	To explore women’s views and experiences in achieving their recommended glycemic treatment targets and to identify potential barriers and enablers.	Cross-sectional	60 European and Asian ethnicity	Mail	Blood glucose monitoring
Kolivand et al, 2018, Iran [41]	To determine the needs of women as an essential first step to formulate a self-care guide fitting the Iranian culture.	Qualitative	13 Iranian	Clinic patients	Diet and exercise
Haghdoost et al, 2019, Iran [42]	To explore the relationship between socioeconomic status and adherence of patients with GDM to medical orders.	Prospective study	230 Iranian women	Clinic patients	Diet, exercise, and blood glucose monitoring
Khalil, 2019, France [43]	To understand, from patients’ and health care professionals’ perspectives, what drives the adoption and diffusion of a telemonitoring solution (myDiabby; MDHC SAS) in a context where telemonitoring activities are still not compensated like traditional follow-ups.	Qualitative	15 women	From the list of patients who had experience of using myDiabby	Diet and blood glucose monitoring
Lawrence et al, 2021, New Zealand [20]	To explore how women diagnosed with GDM perceive dietary recommendations and how this information influences their dietary decisions during pregnancy and beyond.	Qualitative	18 women with a diverse range of ethnic backgrounds	Phone	Diet
Elton, 2022, United Kingdom [44]	To redress a gap in the literature by exploring the intersection between pregnancy and gestational diabetes, and the community that develops therein.	Qualitative	18 women	Online recruitment from a GDMums support group	Diet and exercise
Mukona et al, 2017, Zimbabwe [45]	To explore the barriers to adherence and identify possible solutions to nonadherence to antidiabetic therapy among women with diabetes in pregnancy.	Qualitative	28 women	Clinic patients	Diet and exercise
Kozica-Olenski et al, 2022, Australia [46]	To explore the experiences and acceptability of telehealth use in diabetes in pregnancy care during the COVID-19 pandemic, from the perspectives of pregnant women and their clinicians. The secondary aim was to explore the experiences of pregnant women receiving general maternity care via telehealth during the COVID-19 pandemic.	Qualitative	18 women	Email invitation	Diet, exercise, and blood glucose monitoring
Carolan et al, 2012, Australia [47]	To explore the factors that facilitated or inhibited gestational diabetes self-management among women in a socially deprived area.	Qualitative	15 women with diverse ethnic group	Clinic patients	Diet, exercise, and blood glucose monitoring
Martis et al, 2018, New Zealand [48]	To identify enablers and barriers for women with GDM to achieve optimal glycemic control.	Qualitative	60 women with diverse ethnic group	Email invitation	Blood glucose monitoring
Hewage et al, 2020, Singapore [49]	To understand the perception of patients and health care providers of the barriers to GDM management and preferred interventions to manage GDM in an Asian setting.	Mixed methods	216 women (survey) and 15 women (interview)	Clinic patients	Diet, exercise, and blood glucose monitoring
Bandyopadhyay, 2021, Australia [50]	To gain a better understanding of the lived experiences of South Asian women and their experiences of self-management and their health care providers’ perspectives of treatment strategies.	Qualitative	23 South Asian women	Clinic patients	Diet, exercise, and blood glucose monitoring
Rasekaba et al, 2021, Australia [51]	To identify the profiles of women accessing care for GDM in a large regional hospital with a rural catchment in Victoria, Australia, as well as gain insight into the views of the women with GDM, clinicians, and IT staff on the acceptability and feasibility of a GDM telehealth in this setting.	Qualitative	9 women	Clinic patients	Diet and blood glucose monitoring
Sabag et al, 2023, Australia [52]	To determine how women with GDM manage their condition and to identify the primary supports and barriers to lifestyle intervention participation.	Cross-sectional	564 women with diverse ethnic group	Online recruited through social media	Diet and exercise
Oxlad et al, 2023, Australia [53]	To explore women’s perspectives and experiences concerning how culture impacts the lifestyle management of GDM in women of culturally and linguistically diverse backgrounds.	Qualitative	33 women with diverse ethnic background	Telephoned to invite the penitential participants	Diet
He et al, 2024, China [54]	To gain insight into the experiences of pregnant nurses with GDM in China in terms of their illness, work burdens, and self-care.	Qualitative	9 pregnant nurses	Clinic patients	Diet, exercise, and blood glucose monitoring
Merchant et al, 2025 , United States [55]	To examine patient perspectives on social support’s role in GDM management during pregnancy and early postpartum.	Qualitative	38 women	Clinic patients	Diet, exercise, blood glucose monitoring, and insulin shot
Pham et al, 2025, Australia [56]	To examine women’s experiences of GDM online communities on Facebook, their motivations for participation, and perceptions of dynamics within the community.	Qualitative	28 women	Online recruitment through Facebook	Diet, exercise, blood glucose monitoring, and insulin shot

a GDM: gestational diabetes mellitus.

### Quality Assessment Results

The results, detailed in Multimedia Appendix 1, demonstrate high methodological quality overall. Of the 30 appraised studies, the majority were qualitative (n=24), with quantitative descriptive (n=4) and mixed methods (n=2) studies. The vast majority of criteria were rated “Yes.” A small number of “Cannot tell” ratings were present, primarily for criterion 1.5 (qualitative) and criterion 4.4 (quantitative descriptive). No study received more than 2 such ratings, supporting the robustness of the evidence base.

### Factors Identified as Obstacles and Enablers

#### Overview

Across the 30 included studies, a total of 32 distinct barriers and enablers influencing adherence to GDM self-management were extracted. These were synthesized into 11 overarching factors and mapped to 5 levels of the SEM (Table 2). A detailed list of extracted factors is provided in Multimedia Appendix 1. This synthesis offers an evidence-based framework for understanding the multilevel influences on GDM self-management and provides actionable insights to inform the development of targeted interventions aimed at improving adherence and health outcomes for pregnant women with GDM. The following sections present the identified obstacles and enablers at each SEM level, supported by qualitative findings and triangulated with quantitative data from the included studies.

**Table 2.** Factors identified as obstacles and enablers to adherence to self-management of gestational diabetes mellitus (GDM) from selected studies.

SEM^a^ level and factors		Obstacles	Enablers	Quantitative support
Intrapersonal				
Disease and self-management literacy		Knowledge and language barriers [28,35-39,41,46,47,49,50,53]	Knowledge as empowerment [29,32,33,38,41,44,47,54,56]	Language barrier: 55% reported not having info in first language [40] Knowledge is a key factor for adherence [42]
Emotional and cognitive response		Overwhelmed by diagnosis of GDM [20,29,31-33,38,39,50]	Concern for maternal/fetal health [20,28,31,36,38,47], positive attitude [36,54]	Concern for fetal complications was top motivator for diet (67.1%) and exercise (50%) [34]
Physical capacity and personal logistics		Physical discomfort [28,33,38,45,47,50] and time constraints [28,38,39,47,49,51,54]	—^b^	Frequent hunger: 61.6% [40] Lack of time: 71.4% [52]
Interpersonal				
Functional support network		Absence of support from family [20,28,35,39,41,45,47-49,53,54], lack of shared responsibility for family or work commitments [28,32,37,39,45,49,54], and family or friends providing unhealthy food [55]	Emotional and logistical support [28-30,32,35-38,40,41,45,47-51,53-55], and collaborative healthy meal preparation [55]	Family offering unhealthy food: 38.3% [40] Husband encouragement: 22.6%‐28.7% [34] Family commitment: 32.9%‐40.9% [34]
Organizational				
Quality and applicability of health care		Confusing information [28,33,35,36,40,41,47,49,56] and culturally inappropriate advice [20,29,30,32,33,37,50,53]	Comprehensible guidance [28,29,47,49] and supportive patient-provider relationship [20,35-38,41,45,46,49]	Comprehensible guidance valued by 100% [40] Support from health care providers valued by 68% [40]
Accessibility and continuity of health care		Fragmented care [37,40,44-46,48,51,56] and hard-to-access services [20,29,30,32,33,37,50,53]	Consistent care [31,51] and technology-supported services [17,31,43,46,51]	Fragmented care: inconsistent info (16.6%), never seeing same health care provider twice (13.3%) [40] Online format supported by 62.7% [52]
Workplace demands and accommodations		Shift work or inflexible schedules [54]	Understanding managers and workplace adjustments [54]	—
Community				
Food environment and cultural norms		Limited food choice [28,37,39,44,47,49,53] and cultural food norms [54,55]	—	—
Digital information and peer support landscape		Misinformation of online [30,35]	Trusted online resources [28,30,37,49,54,56] and structured peer support [55,56]	—
Policy				
Regulatory and infrastructural systems		Lack of reimbursement or funding for digital health services [43], and lack of workplace accommodation policies [54]	Shared care models [44,49]	—
Economic support systems		High cost of healthy food and devices [45,46]	Financial aid or complimentary services [45,48]	—

a SEM: socioecological model.

b Not applicable.

#### Intrapersonal Level

Analysis of the included studies identified 3 key factors at the intrapersonal level: disease and self-management literacy, emotional and cognitive responses, and physical capacity and personal logistics.

##### Disease and Self-Management Literacy

A significant obstacle was a deficit in knowledge about GDM and its management, compounded by language barriers, which hindered women’s ability to seek out and understand necessary resources [28,35-39,41,46,47,49,50,53]. Quantitative studies substantiated this; 33 of 60 (55%) women reported that not having information in their first language was a barrier [40], and another study highlighted the critical role of knowledge in adherence [42]. Conversely, acquiring knowledge was perceived by women as a valuable enabler, supporting their confidence and motivation to engage in self-management behaviors [29,32,33,38,41,44,47,54-56]. Nevertheless, the translation of knowledge into sustained behavioral change remained contingent on additional factors across other SEM levels.

##### Emotional and Cognitive Responses

The diagnosis of GDM often provoked overwhelming feelings of panic, stress, and being overwhelmed [20,29,31-33,38,39,50]. A primary enabler to counter these emotional challenges was concern for both maternal and fetal health, which motivated adherence to self-management regimens [20,28,31,36,38,47]. Survey data strongly supported this; concern for fetal complications was the most frequently reported motivator for both diet (67.1%) and physical activity (50%) [34]. Furthermore, maintaining a positive attitude was identified as a key factor in sustaining motivation [36,54].

##### Physical Capacity and Personal Logistics

Physical discomforts, including physical tiredness and frequent hunger, presented substantial obstacles to adhering to diet and exercise recommendations [28,33,38,45,47,50]. This was commonly quantified, with 61.6% (37/60) of women in 1 study reporting frequent hunger as a barrier [40]. Lack of time was the most prevalent barrier in large surveys, reported by 71.4% of women [52].

### Interpersonal Level

The functional support network provided by a woman’s immediate social circle was a pivotal factor influencing GDM self-management. A significant obstacle was the absence of this support from family and partners, which left women without the necessary encouragement and practical help [20,28,35,39,45,47-49,53]. This challenge was often exacerbated when family or friends provided unhealthy food, creating interpersonal conflict and directly undermining dietary adherence [55]. A related obstacle was the lack of shared responsibility for family and work commitments, which placed the burden of managing GDM solely on the woman amidst her existing duties [28,32,37,39,45,49,54]. Quantitatively, 38.3% (23/60) of women identified being offered unhealthy food by family or colleagues as a barrier [40], while family and child care responsibilities were also frequently cited (32.9%‐40.9%) [34].

Conversely, a robust functional support network served as a powerful enabler. This included emotional and appraisal support—such as encouragement, empathy, and shared accountability—which helped women cope with the stress of their diagnosis and maintain motivation [28-30,32,35-38,40,45,48-50,53-55]. Husband encouragement was a key motivator, reported by 22.6% to 28.7% of women [34]. Tangible, indirect logistical aid was equally critical, with collaborative healthy meal preparation by family members being a key factor in successfully managing dietary changes [28-30,32,35-38,40,45,48-50,53].

### Organizational Level

At the organizational level, the structure and delivery of health care services significantly influence self-management. The analysis identified 3 key factors: the quality and applicability of health care, its accessibility and continuity, and workplace demands.

#### Quality and Applicability of Health Care

A primary obstacle was the provision of confusing or insufficient information about GDM management [28,33,35,36,40,41,47,49,56]. This issue was often compounded by culturally inappropriate advice, particularly regarding dietary recommendations, which reduced the practicality of the guidance for women from diverse backgrounds [20,29,30,32,33,37,50,53]. Enablers included receiving comprehensible guidance [28,29,47,49]—a factor universally valued, with 100% (60/60) of women in 1 study endorsing its importance [40]—and having a supportive patient-provider relationship, which fostered trust and improved adherence [20,35-38,41,45,46,49]. Support from health professionals was also highly valued (41/60, 68%) [40].

#### Accessibility and Continuity of Health Care

Women reported frustration with fragmented care and a lack of consistent contact with their health care providers [37,40,44-46,48,51,56]. Services were often hard to access due to long waiting times, insufficient consultation durations, and a lack of out-of-office support [20,29,30,32,33,37,50,53]. Quantitative findings specified that inconsistent information (16.6%) and never seeing the same professional twice (13.3%) were reported barriers [40]. A key enabler was the provision of consistent care [31,51]. Furthermore, these accessibility issues were effectively addressed through technology-supported services, such as telehealth, mobile applications, and online platforms, which provided timely feedback, reliable information, and overcame geographical barriers [17,31,43,46,51]. A majority (62.7%) of women in a large survey supported having an online format for lifestyle interventions [52].

#### Workplace Demands and Accommodations

The demands of a woman’s job also presented an organizational-level obstacle, with challenges such as shift work interfering with the ability to maintain consistent meal times and clinical appointments [54]. An enabler identified was supportive managers and workplace adjustments [54].

### Community Level

At the community level, broader societal and environmental factors play a significant role. The analysis identified 2 key factors: the food environment and cultural norms, as well as the digital information and peer support landscape.

#### Food Environment and Cultural Norms

Women faced obstacles related to limited food choices, particularly when navigating meals outside the home and managing the high cost of healthy foods [28,37,39,44,47,49,53]. In addition, they encountered cultural food norms [54,55], where traditional expectations around eating during pregnancy often conflicted with GDM dietary recommendations, creating significant social and personal challenges [28,37,39,44,47,49,53].

#### Digital Information and Peer Support Landscape

The digital environment presented a dual challenge. A key obstacle was the prevalence of misinformation online, which made it difficult for women to find reliable guidance for GDM self-management [30,35]. Conversely, access to trusted online resources served as a crucial enabler, providing accessible and valuable information [28,30,37,49,54,56]. Beyond information, structured peer support found in organized online groups was also identified as a key enabler, offering a sense of community and shared experience [55,56].

### Policy Level

At the policy level, systemic and regulatory factors were found to significantly influence the resources available for GDM self-management. The analysis identified 2 key factors: regulatory and infrastructural systems, and economic support systems.

#### Regulatory and Infrastructural Systems

A significant obstacle was the lack of reimbursement and funding for digital health services, which limited their integration into standard care and prevented patients from benefiting from them in practice [43]. Another obstacle identified was the lack of workplace accommodation policies for women with GDM [54]. A key enabler to improve care coordination was the implementation of shared care models, which ensure all aspects of GDM management are addressed collaboratively between the woman and her providers, leading to better overall outcomes [44,49].

#### Economic Support Systems

The high cost of healthy food and monitoring devices posed a substantial financial barrier for many women [45,46]. To mitigate this, the provision of financial aid or complimentary services has been identified as a crucial enabler, ensuring that women have necessary resources to effectively manage their condition [45,48].

## Discussion

### Principal Findings

This systematic review, using the SEM, reveals that adherence to GDM self-management is not primarily an issue of individual willpower but a complex systemic challenge. Factors enabling or hindering women exist and interact across all 5 levels of the SEM—from personal emotions to national policy. The key contribution of this synthesis is, therefore, not merely the identification of these multilevel determinants but the critical insight into how they interlock to create a context that can either overwhelm or empower women. Moving beyond a simple listing of barriers and facilitators, the following discussion integrates these findings to critically inform the development of effective, holistic support strategies, with particular attention to the role of digital health solutions.

Our findings align with and extend existing literature on chronic disease self-management, which emphasizes the limitations of an individual-focused approach [24]. The significant burdens reported at the intrapersonal level—emotional distress, knowledge deficits, and logistical constraints—are consistent across studies of GDM [29,33]. However, this review underscores that these personal challenges are frequently exacerbated by challenges at other levels. For instance, intrapersonal anxiety and confusion are often directly amplified by organizational factors such as conflicting information from providers or a lack of continuity of care [28,37]. Similarly, the interpersonal obstacle of unsupportive family environments is linked to community-level cultural norms about food and pregnancy [54,55]. This pattern confirms the core SEM tenet that individual behavior is embedded within and shaped by broader systems, a perspective sometimes underemphasized in GDM management research, which focuses predominantly on educational interventions. The interconnected nature of these challenges calls for equally interconnected solutions.

A critical synthesis of the results points to 2 dominant, cross-level themes: fragmentation of support and information inequity. Fragmentation occurs when care is discontinuous (organizational), social support is absent (interpersonal), and policies fail to coordinate services (policy), leaving women to manage alone. This isolation intensifies intrapersonal stress. Information inequity arises when accessible, trustworthy guidance is scarce. It manifests as a lack of knowledge (intrapersonal), confusing clinical advice (organizational), and an online landscape rife with misinformation (community). These themes are synergistic, creating a vicious cycle that traps women. For example, a woman may receive vague dietary advice from a busy clinic (organizational), seek clarity online only to encounter conflicting information (community), feel increased anxiety and guilt (intrapersonal), and lack a supportive partner to help her navigate these challenges (interpersonal). Effective interventions must, therefore, be designed to bridge these fragments and curate reliable information flows. The relevance of the SEM for understanding GDM-related behaviors is further supported by recent systematic reviews applying the SEM to postpartum physical activity, which similarly identified critical barriers and facilitators at social, organizational, and community levels beyond the individual [57,58].

Consequently, to disrupt these cycles, support strategies must be multipronged and explicitly designed to counteract these systemic failures. Digital health platforms are uniquely positioned to integrate solutions across SEM levels, but their design must be informed by this nuanced understanding. For example, to combat information inequity, a platform must provide centralized, evidence-based, and culturally tailored education (addressing intrapersonal and organizational failures) while incorporating algorithmically vetted content and professional moderation to create a trusted digital community (addressing community-level risks). To address fragmentation of support, features could include shared care plans visible to both patients and providers to improve continuity (organizational), tools to involve and educate family members (interpersonal), and integrated telehealth functions that bypass geographical and time barriers (organizational or community). Crucially, for such tools to be equitable, sustainable, and trustworthy, policy-level advocacy for reimbursement, funding, and robust data security and privacy protection frameworks is essential [43].

These design principles require special consideration for high-risk or underserved populations identified in this review. A critical finding is that challenges related to understanding information should not be reductively classified merely as “language barriers.” For women with limited health literacy—who struggle to comprehend complex medical information even in their primary language—and for working professionals, the health care system’s responsibility extends beyond translation. Providers must prioritize simplifying information, using clear communication, and ensuring cultural relevance to actively improve health literacy. For employed women, such as nurses [54], asynchronous, flexible digital features that accommodate shifting schedules are critical. A one-size-fits-all tool risks perpetuating the very inequities it seeks to solve.

This review has several limitations. Although the methodological quality of the included studies, assessed using the MMAT, was generally high (Multimedia Appendix 1), the preponderance of qualitative studies and those from high-income countries may affect the transferability of findings. The predominance of qualitative studies enriches the understanding of lived experience but limits quantitative generalizability. Furthermore, most included studies were from English-speaking and high-income countries, which may not capture the full spectrum of contextual barriers in low-resource settings and may affect the transferability of findings. Finally, while the SEM provided a valuable organizing framework, the classification of some factors (eg, personal logistics) into a single level is inherently somewhat reductive, as influences often span multiple levels.

Supporting GDM self-management, therefore, demands a systemic view. Achieving meaningful improvement will depend on integrating strategies that simultaneously empower the individual, engage her support network, streamline and humanize health care delivery, foster supportive communities, and advocate for enabling policies. Future intervention research, particularly in digital health, must therefore evaluate not just patient-level outcomes but also how effectively solutions address these interconnected, multilevel determinants to achieve truly comprehensive care.

### Implications

The findings of this review underscore the necessity for comprehensive, multilevel strategies to enhance adherence to GDM self-management. These implications target various stakeholders across the socioecological spectrum.

#### Intrapersonal Level

Health care providers must move beyond simple information delivery to provide education that actively increases GDM knowledge and self-efficacy. This should be coupled with psychological support to help women manage the overwhelming feelings often associated with a diagnosis, as well as practical strategies to mitigate physical challenges like frequent hunger and tiredness, which are significant reported barriers.

#### Interpersonal Level

As social support is a pivotal enabler, providers should actively facilitate the involvement of partners and family. Educating the support network can transform it from a potential source of conflict (eg, offering unhealthy food) into a source of essential emotional and practical assistance, with spousal encouragement being a key motivator.

#### Organizational Level

Health care institutions are responsible for ensuring the provision of clear, comprehensive, and culturally sensitive guidelines. To overcome traditional barriers like long waiting times and fragmented care, services should integrate technology, such as telehealth, mobile apps, digital tools, and digital monitoring tools to improve accessibility and continuity—an approach strongly supported by women in need.

#### Community Level

Community services and public health initiatives should develop and promote accessible resources, including practical food-choice guides and vetted online platforms. Fostering structured peer support networks can also provide invaluable social support and shared experiential knowledge for women managing GDM.

#### Policy Level

Policymakers play a crucial role in creating an enabling environment. This includes allocating funding to establish and reimburse digital health services, making them a sustainable part of standard care. Financial subsidies for healthy foods and monitoring devices are essential for equity. Finally, formalizing shared care models through policy can reinforce a collaborative, system-wide approach to GDM management.

### Conclusions

In conclusion, this systematic review demonstrates that successful self-management of GDM requires an integrated, multilevel approach informed by the SEM. Moving beyond a focus on individual responsibility, effective support must simultaneously address intrapersonal, interpersonal, organizational, community, and policy factors. By designing interventions and systems that acknowledge and bridge these interconnected levels, health care providers, policymakers, and communities can significantly improve adherence to self-management, ultimately leading to better health outcomes for mothers and their children.

## Supplementary material

10.2196/86767 Multimedia Appendix 1 Mixed Methods Appraisal Tool (MMAT) quality appraisal results.

10.2196/86767 Checklist 1 PRISMA 2020 checklist.

## Abbreviations

GDM: gestational diabetes mellitus
MMAT: Mixed Methods Appraisal Tool
PRISMA: Preferred Reporting Items for Systematic Reviews and Meta-Analyses
SEM: socioecological model
