# Supplementary material for: Obstacles and Enablers Related to Gestational Diabetes Self-Management: Systematic Review Using the Socio-Eecological Model
Source: JMIR Diabetes. 2026 Jun 1;11:e86767. doi: 10.2196/86767 (PMC13225225; doi:10.2196/86767)
Supplement: Multimedia Appendix 1 [file diabetes-v11-e86767-s001.docx]

| **Supplementary Table Mixed Methods Appraisal Tool (MMAT) quality appraisal results** | | | | | | | | | | | | | | | |
| --- | --- | --- | --- | --- | --- | --- | --- | --- | --- | --- | --- | --- | --- | --- | --- |
| **[Ref. No. of the studies]** | **Qualitative** | | | | | **Quantitative** | | | | | **Mix-methods** | | | | |
|  | 1.1 | 1.2 | 1.3 | 1.4 | 1.5 | 4.1 | 4.2 | 4.3 | 4.4 | 4.5 | 5.1 | 5.2 | 5.3 | 5.4 | 5.5 |
| **[37]** | Y | Y | Y | Y | Y |  |  |  |  |  |  |  |  |  |  |
| **[42]** | Y | Y | Y | Y | Y |  |  |  |  |  |  |  |  |  |  |
| **[54]** | Y | Y | Y | Y | Y |  |  |  |  |  |  |  |  |  |  |
| **[48]** | Y | Y | Y | Y | C |  |  |  |  |  |  |  |  |  |  |
| **[43]** | Y | Y | Y | Y | Y |  |  |  |  |  |  |  |  |  |  |
| **[44]** | Y | Y | Y | Y | Y |  |  |  |  |  |  |  |  |  |  |
| **[49]** |  |  |  |  |  | Y | Y | Y | C | Y |  |  |  |  |  |
| **[28]** | Y | Y | Y | Y | Y |  |  |  |  |  |  |  |  |  |  |
| **[30]** |  |  |  |  |  |  |  |  |  |  | Y | Y | Y | Y | Y |
| **[31]** | Y | Y | Y | Y | Y |  |  |  |  |  |  |  |  |  |  |
| **[29]** | Y | Y | Y | Y | Y |  |  |  |  |  |  |  |  |  |  |
| **[32]** | Y | Y | Y | Y | C |  |  |  |  |  |  |  |  |  |  |
| **[40]** |  |  |  |  |  | Y | Y | Y | C | Y |  |  |  |  |  |
| **[33]** | Y | Y | Y | Y | C |  |  |  |  |  |  |  |  |  |  |
| **[41]** |  |  |  |  |  | Y | Y | Y | C | Y |  |  |  |  |  |
| **[56]** | Y | Y | Y | Y | Y |  |  |  |  |  |  |  |  |  |  |
| **[20]** | Y | Y | Y | Y | Y |  |  |  |  |  |  |  |  |  |  |
| **[45]** | Y | Y | Y | Y | C |  |  |  |  |  |  |  |  |  |  |
| **[50]** | Y | Y | Y | Y | Y |  |  |  |  |  |  |  |  |  |  |
| **[38]** | Y | Y | Y | Y | Y |  |  |  |  |  |  |  |  |  |  |
| **[34]** | Y | Y | Y | Y | Y |  |  |  |  |  |  |  |  |  |  |
| **[53]** | Y | Y | Y | Y | Y |  |  |  |  |  |  |  |  |  |  |
| **[35]** |  |  |  |  |  |  |  |  |  |  | Y | Y | Y | Y | Y |
| **[36]** | Y | Y | Y | Y | Y |  |  |  |  |  |  |  |  |  |  |
| **[51]** | Y | Y | Y | Y | C |  |  |  |  |  |  |  |  |  |  |
| **[52]** |  |  |  |  |  | Y | Y | Y | Y | Y |  |  |  |  |  |
| **[39]** | Y | Y | Y | Y | Y |  |  |  |  |  |  |  |  |  |  |
| **[47]** | Y | Y | Y | Y | Y |  |  |  |  |  |  |  |  |  |  |
| **[46]** | Y | Y | Y | Y | Y |  |  |  |  |  |  |  |  |  |  |
| **[55]** | Y | Y | Y | Y | Y |  |  |  |  |  |  |  |  |  |  |

Y=Yes, N=No, C= Can’t tell
